# Meta-analysis to compare the accuracy of GeneXpert, MODS and the WHO 2007 algorithm for diagnosis of smear-negative pulmonary tuberculosis

**DOI:** 10.1186/1471-2334-13-507

**Published:** 2013-10-30

**Authors:** Simon Walusimbi, Freddie Bwanga, Ayesha De Costa, Melles Haile, Moses Joloba, Sven Hoffner

**Affiliations:** 1 Department of Medical Microbiology, Makerere University, Kampala, Uganda; 2 Department of Public Health Sciences, Karolinska Institute, Stockholm, Sweden; 3 Department of Diagnostics and Vaccinology, Swedish Institute for communicable Disease Control, Solna, Sweden

**Keywords:** Smear negative, Pulmonary TB, GeneXpert, MODS, WHO TB algorithm

## Abstract

**Background:**

Smear-negative pulmonary tuberculosis (SN-PTB), which is common in HIV-infected patients, is difficult to diagnose using smear microscopy alone. In 2007, the WHO developed an algorithm to improve the diagnosis and management of smear-negative tuberculosis in HIV prevalent and resource constrained settings. Implementation of the algorithm required individuals with presumptive TB to be initially evaluated using two sputum microscopy examinations followed by clinical diagnosis that may include chest X-ray and antibiotic treatment in smear-negative individuals. Since that time, the WHO has endorsed several new tests for diagnosis of tuberculosis. However, it is unclear how the new tests perform when compared to the WHO 2007 algorithm in diagnosis of SN-PTB. Using meta-analysis study design, we summarized and compared the accuracy of Xpert® MTB/Rif assay (GeneXpert) and Microscopic Observation Drug Susceptibility assay (MODS), with the WHO 2007 algorithm in the diagnosis of SN-PTB.

**Methods:**

A systematic review and meta-analysis of publications on GeneXpert, or MODS, or the WHO 2007 algorithm for diagnosis of SN-PTB, using culture as reference test was performed. Meta-Disc software was used to obtain pooled sensitivity and specificity of the diagnostic methods. Heterogeneity in the accuracy estimates was tested by reviewing the generated forest plots, sROC curves and the Spearman correlation coefficient of the logit of true positive rate versus the logit of false positive rate.

**Results:**

Twenty-four publications on all three diagnostic methods were meta-analyzed. The pooled sensitivity and specificity for detection of smear-negative pulmonary tuberculosis were 67% and 98% for GeneXpert, 73% and 91% for MODS, and 61% and 69% for WHO 2007 algorithm, respectively. The sensitivity of GeneXpert reduced from 67% to 54% when sub-group analysis of studies with patient HIV prevalence ≥30% was performed.

**Conclusion:**

The GeneXpert, MODS, and the WHO algorithm have moderate to high accuracy for the diagnosis of SN-PTB. However, the accuracy of the tests is extremely variable. The setting and context under which the tests are conducted in addition to several other factors could explain this variability. There is therefore need to investigate these factors further. The information from these studies would inform the adoption and placement of these new tests.

## Background

The global burden of tuberculosis (TB) remains high with 8.7 million new TB cases estimated to have occurred in 2012 [1]. The majority of the new TB cases (80%) occurred in 22 countries and a substantial proportion (35%) were smear-negative pulmonary TB (SN-PTB). In these countries, TB diagnosis relies mainly on smear microscopy which has a highly variable sensitivity ranging from 20% to 60% [2,3]. In sub Saharan Africa, where the prevalence of 44 HIV is relatively high and TB is a common opportunistic 45 infection, TB/HIV co-infected patients frequently present with SN-PTB. This is because HIV patients usually form poor lung granulomas/cavities when infected with TB, resulting in lower concentrations of Mycobacterium tuberculosis (Mtb) in the lesions [4], which can pose diagnostic difficulties [5].

**Figure 1:**
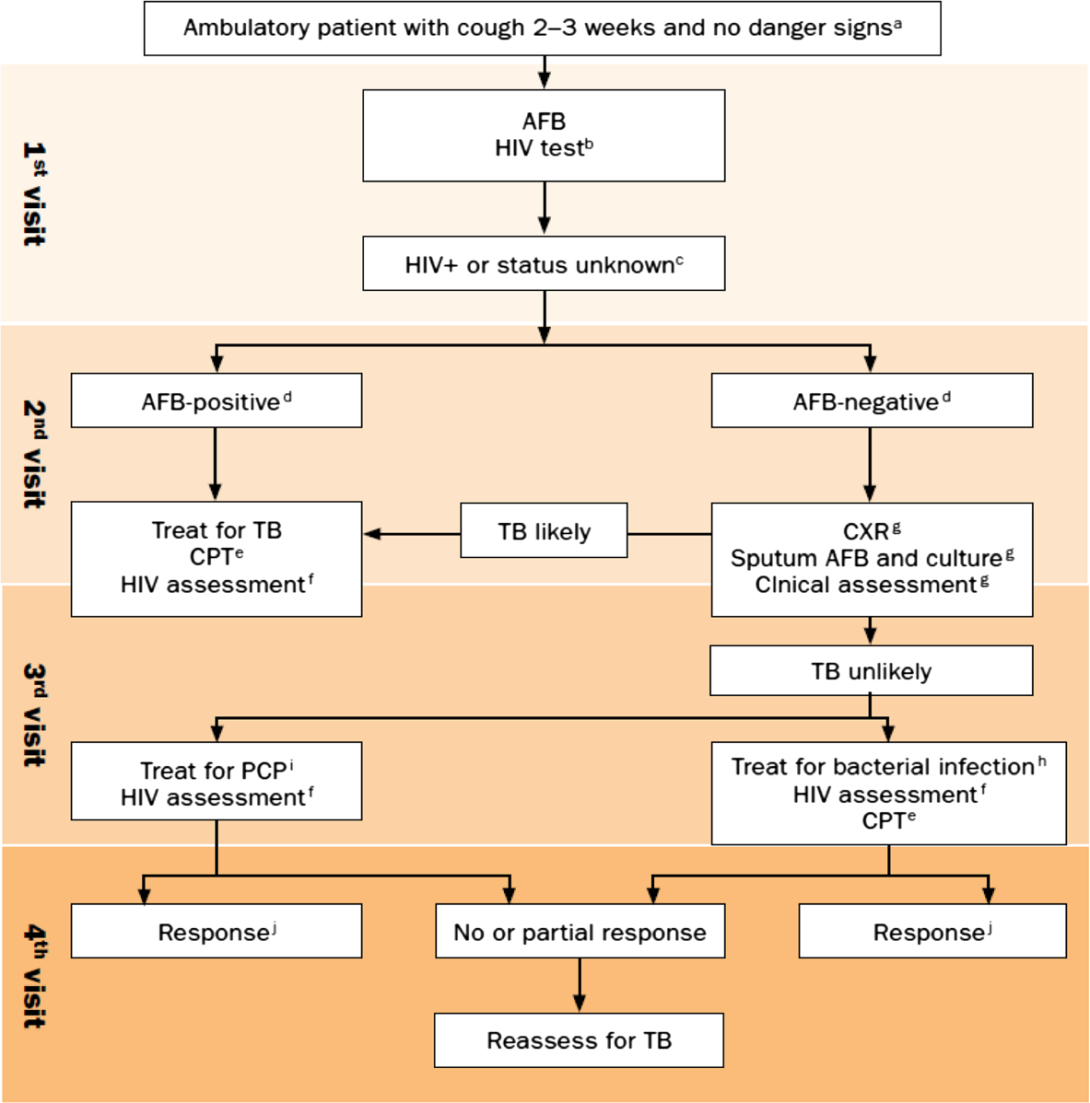
**WHO 2007 algorithm for the diagnosis of TB in ambulatory HIV-positive patients.** a) the danger signs include any one of: respiratory rate > 30/minute, fever > 39°C, pulse rate > 120/min and unable to walk unaided. b) for countries with adult HIV prevalence rate = 1% or prevalence rate of HIV among tuberculosis patients = 5%. c) In the absence of HIV testing, classifying HIV status unknown as HIV-positive depends on clinical assessment or national and/or local policy. d) AFB-positive is defined at least one positive and AFB-negative as two or more negative smears. e) CPT = Co-trimoxazole preventive therapy. f) HIV assessment includes HIV clinical staging, determination of CD count if available and referral for HIV care. g) the investigations within the box should be done at the same time wherever possible in order to decrease the number of visits and speed up the diagnosis. h) antibiotics (except fluoroquinolones) to cover both typical and atypical bacteria should be considered. i) PCP: *Pneumocystis carinii* pneumonia, also known as *Pneumocystis jirovecii* pneumonia*.* j) advise to return for reassessment if symptoms recur.

In 2007, the WHO issued an algorithm for the diagnosis of SN-PTB for use in resource-limited settings with high HIV infection rates [6]. Adoption of this algorithm (Figure 1), was expected to improve diagnosis and management of smear-negative tuberculosis. However, the diagnostic methods used when the algorithm was made, have since then been improved upon or entirely new tests have been developed. The WHO has also endorsed several of these new tests [7]. Further, the WHO 2007 algorithm outlines a lengthy diagnostic pathway which requires a patient to visit the clinic four times before a clinician decides whether to treat a patient as a case of smear-negative tuberculosis. In practice, few patients complete all the elements of the algorithm (see Figure 1) before a decision to treat or not is taken [8]. In addition, although the algorithm encourages sputum culture during the second clinic visit to assist the confirmation of diagnosis of smear-negative TB, this is often not practically possible. Reasons for this include firstly that the commonly available TB culture method in many of the focus settings is the Lowenstein-Jensen (LJ) method, a solid based medium that takes several weeks to detect bacterial growth. Secondly, in many of the countries for which the algorithm was developed, culture facilities are often limited to reference laboratories with insufficient capacity to meet the national demand for culture confirmation [1]. Because of the reasons mentioned above among others, there has been limited success in improving diagnosis of smear-negative TB using the algorithm.

More recently, the WHO endorsed the Xpert® MTB/Rif assay (GeneXpert) for the diagnosis of TB [9]. The GeneXpert relies on DNA-PCR technique for detection of TB and Rifampicin resistance related mutations simultaneously. It is the first molecular assay for TB detection to be fully automated and to integrate all the steps required for PCR-based DNA test. It gives results within 3 hours. The test has also been reported to be highly accurate for diagnosis of pulmonary TB [10]. Patients with presumptive HIV-associated TB who are negative on smear examination are the most likely to benefit from GeneXpert [11].

Another new test is the Microscopic Observation Drug Susceptibility assay (MODS). The WHO recently endorsed the test for rapid screening of multidrug-resistant TB [12]. The MODS relies on two well-known properties of M.tb i.e. (i) the rate of growth of M.tb in liquid medium is considerably quicker than on solid medium(ii) the morphology of M.tb in liquid culture is characteristic and recognizable, consisting of so called “cord” like structures [13]. Thus by using an inverted light microscope to examine tissue culture plates inoculated with sputum, M.tb growth can be detected within 7–10 days, compared to conventional solid culture that takes several weeks [14]. In settings where conventional culture services for diagnosis of TB are not readily available, the MODS could be an alternative for early diagnosis of SN-PTB since it is simple, rapid and cheap.

However, evidence on the performance of the GeneXpert, MODS assay, and the WHO 2007 algorithm for diagnosis of SN-PTB is scanty. In this study, we did a meta-analysis to summarize and compare the accuracy (sensitivity and specificity) of the GeneXpert (a molecular based assay), the MODS (a rapid culture method) and the WHO 2007 algorithm (an algorithm based method) for the diagnosis of SN-PTB. We considered all the elements of the WHO 2007 algorithm (its entirety) as one test.

## Methods

### Study design

A systematic review of publications on GeneXpert, MODS and the WHO 2007 algorithm for the diagnosis of SN-PTB was performed, followed by a meta-analysis.

### Search strategy

Initially, we performed an electronic search in Pubmed without year restriction for articles in English for each test individually. The search terms used were ‘GeneXpert’, ‘Microscopic observation drug susceptibility’, and ‘WHO TB algorithm’. We then reviewed the retrieved abstracts and selected publications for full text review. After fully reading the selected publications, their bibliographies were also reviewed and relevant additional publications were also retrieved for full text review. To ensure that no relevant publications were missed, we also performed a search in Google Scholar, but no additional publications were found.

### Inclusion

We selected peer-reviewed articles published until 30th May 2012. The publications should have used the GeneXpert, or MODS, or WHO 2007 algorithm, for diagnosis of pulmonary TB. The inclusion criteria were: i) use of culture as the reference method (LJ, or 7H10 agar, or BACTEC 460, or BACTEC MGIT 960). ii) Publications should have reported data to allow first hand computation of sensitivity and specificity of the test for SN-PTB. In papers where this was not reported, we contacted the corresponding authors to request provision of the required data.

### Data extraction

We created an excel spreadsheet and collected data on 20 variables per article, including: index test, author and year of publication, culture method, country of study, study HIV prevalence, sample size, specimen type, culture method, and numbers of true positive, true negative, false positive, and false negative. Numbers of the positive and negative values were extracted either directly or through calculation based on reported measures of accuracy. The obtained data were verified by a second investigator.

### Assessment of quality of study publications

Publications included in the meta-analysis were assessed for quality using the QUADAS-2 tool [15]. The tool consists of four key domains that judge bias and applicability of the reviewed studies by reviewing how patients were selected, the index test, the reference standard, and the flow of patients through the study. These variables were also included in the main data excel spreadsheet.

### Data analysis

From the main spreadsheet we created sub files for GeneXpert, MODS and the WHO 2007 algorithm. Each file was configured to fit into the Meta-Disc software v.1.4 for data analysis [16]. Using the random-effects model, the accuracy of each diagnostic method was analyzed and presented in form of forest plots. We used the forest plots to obtain a general overview of the accuracy estimates of each study before subsequent interpretation of the pooled summary estimates. Sensitivity was defined as the proportion of positive results obtained while specificity was defined as the proportion of negative results obtained, for each diagnostic method in reference to culture. For one of the publications on the WHO 2007 algorithm [8], we analyzed and reported the results separately. This is because the authors aimed to evaluate the effect of various patient and provider factors on the performance of the algorithm in a rural versus urban setting. They therefore reported the diagnostic performance of the algorithm at the two sites separately but in one publication.

### Analysis for heterogeneity

As study results can be variable (heterogeneous), it is critical to explore this heterogeneity to understand the possible factors that influence the obtained accuracy estimates and whether it is appropriate to pool them. Heterogeneity can either be due to chance or due to differences in the threshold that is used to define positive and negative results of a test.

We explored for heterogeneity due to chance (other than threshold effect) for each diagnostic method by; i) visual inspection of the forest plots for deviation of sensitivity and specificity of each study from the vertical line corresponding to the pooled estimates. Large deviations from this line would indicate possibility of heterogeneity, (ii) Chi-square *p*-values, which are automatically computed by Meta-disc during analysis. A low Chi-square p-value would suggest presence of heterogeneity beyond what could be expected by chance alone and, (iii) the inconsistence index (I-square), which is also automatically computed by Meta-disc software. The inconsistence index is a quantitative measure of the amount of heterogeneity [17]. We interpreted the inconsistence index as follows: 0% to 40%: not important; 50% to 70%: represented moderate heterogeneity; > 70% represented substantial heterogeneity [18].

Heterogeneity due to threshold effect was explored by plotting summary receiver operating curves (sROC) for each diagnostic method to assess if the points in the plots had a curvilinear (shoulder arm) pattern or not. A typical “shoulder arm” pattern would suggest presence of threshold effect [16,19,20]. The Meta-disc software automatically computes and shows the statistical analysis of the area under the sROC curve and the Cochrane indices (Q*). As a further assessment of threshold effect, we also calculated the Spearman correlation coefficient between sensitivity (logit of the true positive rate) and specificity (logit of the false positive rate) for each test. If threshold effect exists, an inverse correlation appears. We considered a positive Spearman correlation coefficient of > 0.6 to be strong, and suggestive of threshold effect [21]. If the value was less than 0.6, the accuracy of the tests could be based on pooled estimates of sensitivity and specificity.

## Results

### Publications retrieved

**Figure 2:**
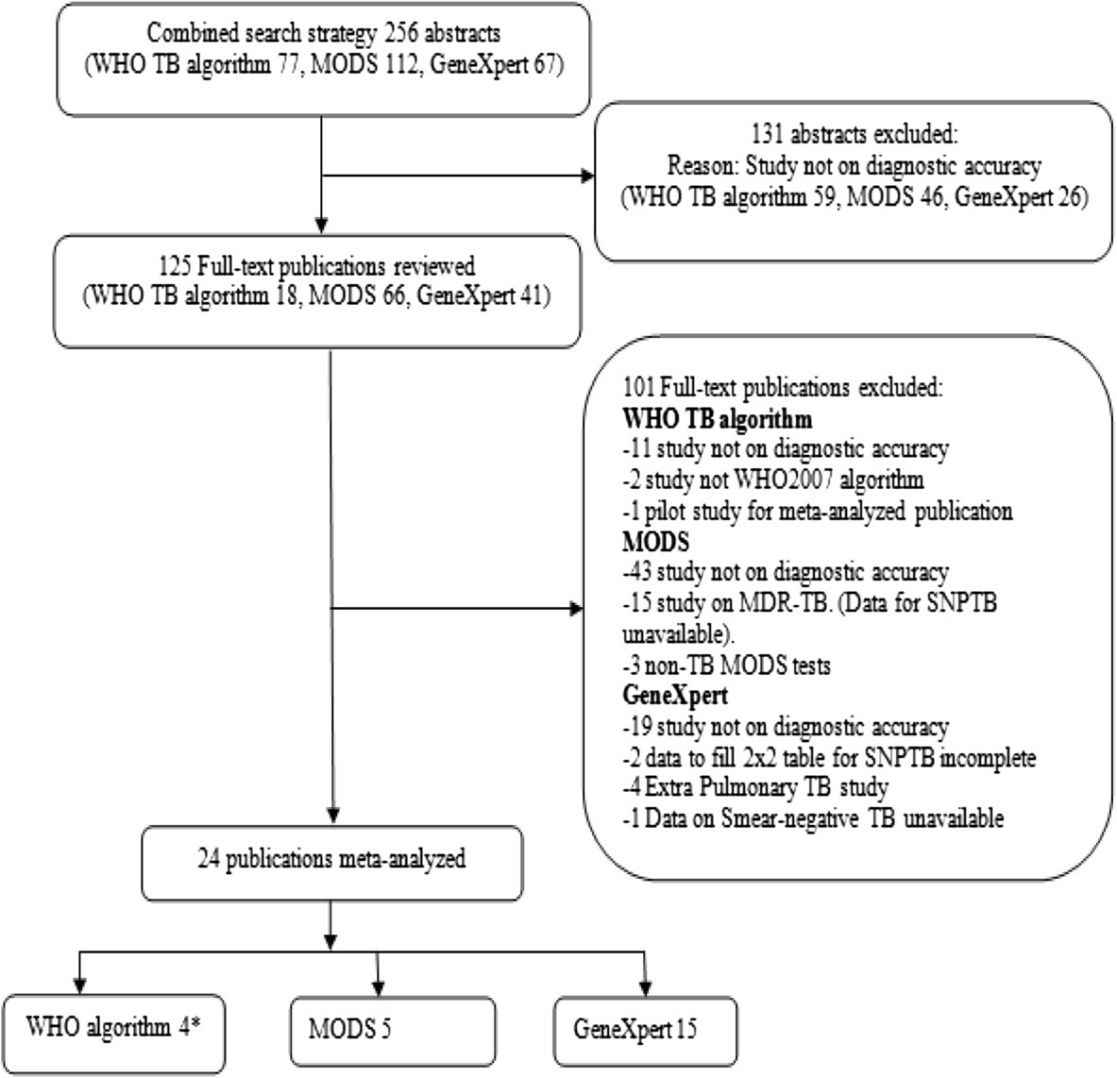
**Flow chart for publication search.** * One of the WHO publications provided separate diagnostic accuracy results for a rural and urban site. The results were therefore reported separately in Table 1. SNPTB = Smear-negative Pulmonary TB. Two landmark studies on GeneXpert were excluded [22,23]. We contacted the author but the data provided remained incomplete to fill 2x2 tables for smear-negative PTB (SNPTB).

The systematic review based on all the stated strategies retrieved a total of 256 abstracts. After reviewing the abstracts, 125 publications (WHO algorithm 18, MODS 66 and GeneXpert 41) were fully reviewed. Due to various reasons such as; a test not being evaluated for diagnostic accuracy or data to allow computation of sensitivity and specificity not reported (see Figure 2), 101 publications were excluded leaving twenty-four publications for final meta-analysis (GeneXpert-15, MODS-5, and WHO 2007 algorithm-4).

### Description of meta-analyzed publications

Of the 24 publications that fulfilled the inclusion criteria for meta-analysis, the study HIV prevalence in 9 of them was ≥50% (GeneXpert-4, MODS-2, WHO 2007 algorithm-3). In addition, 10 of the 24 publications were conducted in countries from sub-Saharan Africa (GeneXpert-6, MODS-1, and WHO 2007 algorithm-3). Further, 6 out of 15 publications on GeneXpert used fluorescent microscopy (FM) as the screening test, while 3 out of 5 publications on MODS used Ziehl-Nielsen microscopy (ZN) as the screening test, and 2 out of 4 publications on the WHO 2007 algorithm used either FM or ZN. A summary of the description of the studies meta-analyzed is presented in Table 1.

**Table 1:** Key characteristics of the meta-analyzed reports (n = 24)

**Test**	**Author, (Year)**	**Ref**	**Country**	**Study HIV rate**	**Specimen type**	**Screen test**	**TP**	**FP**	**FN**	**TN**
GeneXpert	Helb, 2010	[24]	Vietnam	1	Sputum frozen	Unclear	38	0	15	25
	Malbruny, 2011	[25]	France	3.4	Various	FM	6	0	0	73
	Bowles, 2011	[26]	Netherlands	NR	Sputum	ZN	21	0	4	23
	Moure, 2011	[27]	Spain	NR	Sputum frozen	FM + ZN	61	0	17	20
	Marlowe, 2011	[28]	USA	NR	Sputum sediment	Unclear	43	0	12	47
	Theron, 2011	[29]	S. Africa	27	Sputum	FM	22	19	25	319
	Rachow, 2011	[30]	Tanzania	59.9	Sputum frozen	ZN	11	1	7	102
	Scott, 2011	[31]	S. Africa	70*	Sputum sediment	FM	11	3	7	104
	Lawn, 2011	[32]	S. Africa	100	Sputum	FM	23	2	30	320
	Ioannidis, 2011	[33]	Greece	NR	Sputum	Unclear	29	2	3	32
	Miller, 2011	[34]	USA	NR	Sputum frozen	FM	3	2	2	58
	Teo, 2011	[35]	Singapore	NR	Various	ZN	13	2	6	42
	Nicol, 2011	[36]	S. Africa	24	Sputum-induced	FM	25	0	18	166
	Rachow, 2012	[37]	Tanzania	51.2	Sputum	ZN	14	0	7	22
	Safianowska, 2012	[38]	Poland	NR	Various	ZN	4	0	4	181
	**Total**						**324**	**31**	**157**	**1534**
MODS	Arias, 2007	[39]	Brazil / Honduras	12*	Various	ZN	75	28	8	469
	Mashta, 2011	[40]	India	NR	Sputum	ZN	17	45	27	146
	Shah, 2011	[41]	S. Africa	87	Sputum	Unclear	36	13	14	407
	Ha DT, 2010	[42]	Vietnam	100	Sputum	ZN	40	0	15	67
	Chaiyasirinroje, 2012	[43]	Thailand	NR	Sputum	Unclear	13	1	4	37
	**Total**						**181**	**87**	**68**	**1126**
WHO 2007 algorithm	Wilson, 2011	[44]	S. Africa	57*	Sputum-induced	FM	47	91	12	71
	Swai, 2011	[45]	Tanzania	68.1	Sputum	ZN	66	107	61	179
	Koole, 2012	[46]	Cambodia	26.5	Sputum	FM	20	70	14	270
	Alamo, 2012. ^Rural site^	[8]	Uganda	100	Sputum	ZN	18	2	1	1
	Alamo, 2012. ^Urban site^	[8]	Uganda	100	Sputum	ZN	9	13	1	10
	**Total**						**160**	**283**	**89**	**531**

Specimen type various included = bronchial aspirate, bronchial alveolar lavage.

ZN = Ziehl-Nielsen microscopy stain method.

FM = Fluorescent microscopy stain method.

TP = True positive (Individuals have disease and have positive test).

FP = False positive (Individuals do not have disease, but have positive test).

FN = False negative (Individuals have disease, but have negative test).

TN = True negative (Individuals do not have disease and have negative test).

NR = Not reported.

* = The rate reported was based on a denominator that included patients with undocumented HIV result.

### Results on diagnostic accuracy

**Figure 3:**
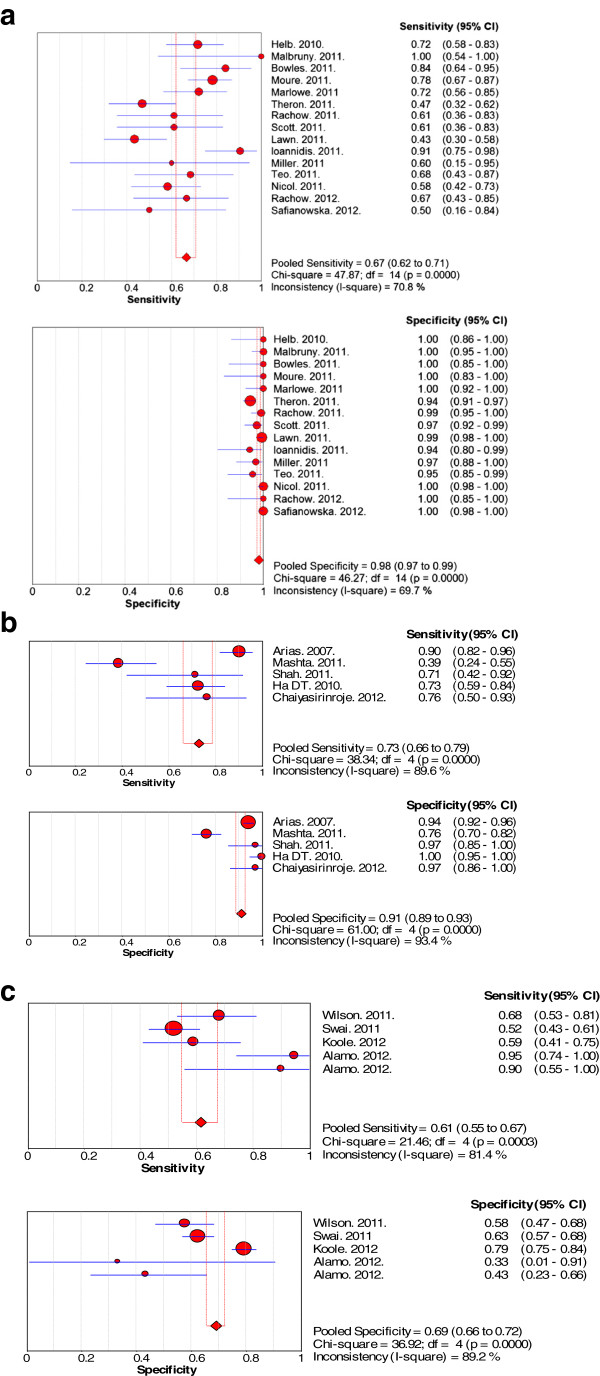
Forest plots of sensitivity and specificity for (a) GeneXpert test, (b) MODS test and (c) WHO 2007 algorithm.

The results of the sensitivity and specificity of each test are shown in Figure 3. Overall, there was large deviation from the pooled estimates in the forest plots for all the three tests indicating the possibility of heterogeneity. However, the deviation was seen more with forest plots for sensitivity than specificity. The Chi-square p-values for heterogeneity for all three tests were low.

#### GeneXpert

The pooled sensitivity and the 95% confidence interval for GeneXpert was 67% (62% to 71%) while the pooled specificity was 98% (97% to 99%). On visualization of the forest plots, there was large deviation from the pooled estimate for sensitivity by several studies. For specificity, deviation from the pooled estimate was small. However, the I-square values for both sensitivity and specificity were above 40%.

#### MODS

The pooled sensitivity and the 95% confidence interval for the MODS test was73% (66% to 79%) while the pooled specificity was 91% (92% to 96%). On visualization of the forest plots, there was large deviation from the pooled estimate for sensitivity by two studies. For specificity, large deviation from the pooled estimate observed for one study. The I-square values for both sensitivity and specificity were above 70%.

#### WHO 2007 algorithm

The pooled sensitivity and the 95% confidence interval for the WHO 2007 algorithm was 61% (55% to 67%) while the pooled specificity was 69% (66% to 72%). On visualization of the forest plots, there was large deviation from the pooled estimate for both sensitivity and specificity by two studies. The I-square values for both sensitivity and specificity were also above 70%.

### Analysis for threshold effect by summary receiver operating curves (sROC)

**Figure 4:**
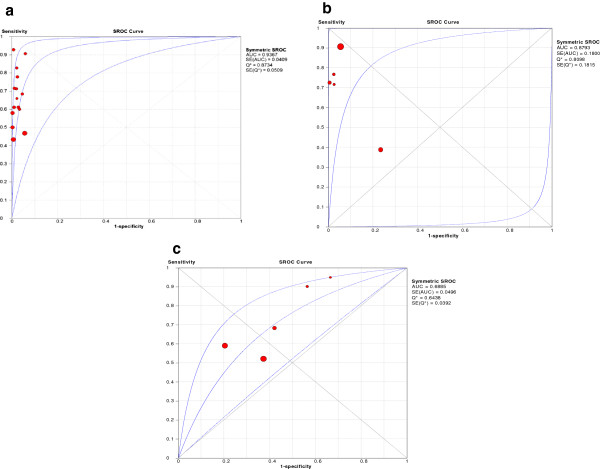
**Summary receiver characteristics (sROC) (a) curve- GeneXpert, (b) curve- MODS and (c) curve- WHO 2007 algorithm.***Note:* sROC = summary receiver operating characteristic curve, which is a plot of the true positive rate (sensitivity) against the false positive rate (1-specificity) of a diagnostic test at different thresholds [47]. This generates a composite statistic (AUC or the Index Q*) that provides an overall evaluation of the accuracy of a test (perfect discriminating ability of true positivity from false positivity). The three curves of the sROC represent the estimate and the 95% upper and lower bounds of the estimate. AUC = Area under the curve of a constructed sROC curve. An AUC close to 1.0 signifies that the test has almost perfect discrimination while an AUC close to 0.5 suggests poor discrimination. An AUC significantly less than 0.5 would indicate that the criteria for “normal” and “abnormal” should be reversed. SE (AUC) = standard error of the area under curve Q* = An index which corresponds to the upper most point on the sROC curve at which sensitivity equals specificity. The closer this value is to 1, the closer the test to perfect accuracy (perfect discriminating ability of true positivity from false positivity). When the value of the Q* index is close to 0.5, it signifies that the test has poor discrimination. SE (Q*) = the standard error of the index Q*.

The patterns of the sROC curves are shown in Figure 4. The curves were consistent with each of the included reports of accuracy, with one outlier point (study) clearly detected in the sROC curve for MODS. The areas under the sROC curves and Cochrane (Q*) indices were 0.94 and 0.87 for GeneXpert, 0.87and 0.81 for MODS, 0.69 and 0.64 for WHO 2007 algorithm, respectively.

### Spearman rank correlation for analysis of threshold effect

The Spearman rank correlations between the logistic transformations (logit) of the true positive rate (TPR) plotted against the logit of the false positive rate (FPR) for each method is presented in Table 2. Only the WHO 2007 algorithm had a significant and strong positive correlation coefficient of threshold effect.

**Table 2:** Spearman correlation coefficient of the logit of TPR versus logit of FPR

**Test**	**Spearman correlation coefficient**	**p- value**
GeneXpert	0.232	0.405
MODS	0.4	0.600
WHO 2007 algorithm	0.9	0.037

Note: The logit of the true positive rate is the natural log of [true positive rate/(1-true positive rate)]. The logit of the false positive rate is the natural log of [false positive rate/(1-false positive rate)].

### Sub-group analysis

Having found indication of possible heterogeneity, we performed the following sub-group analyses.

#### Based on HIV prevalence

**Figure 5:**
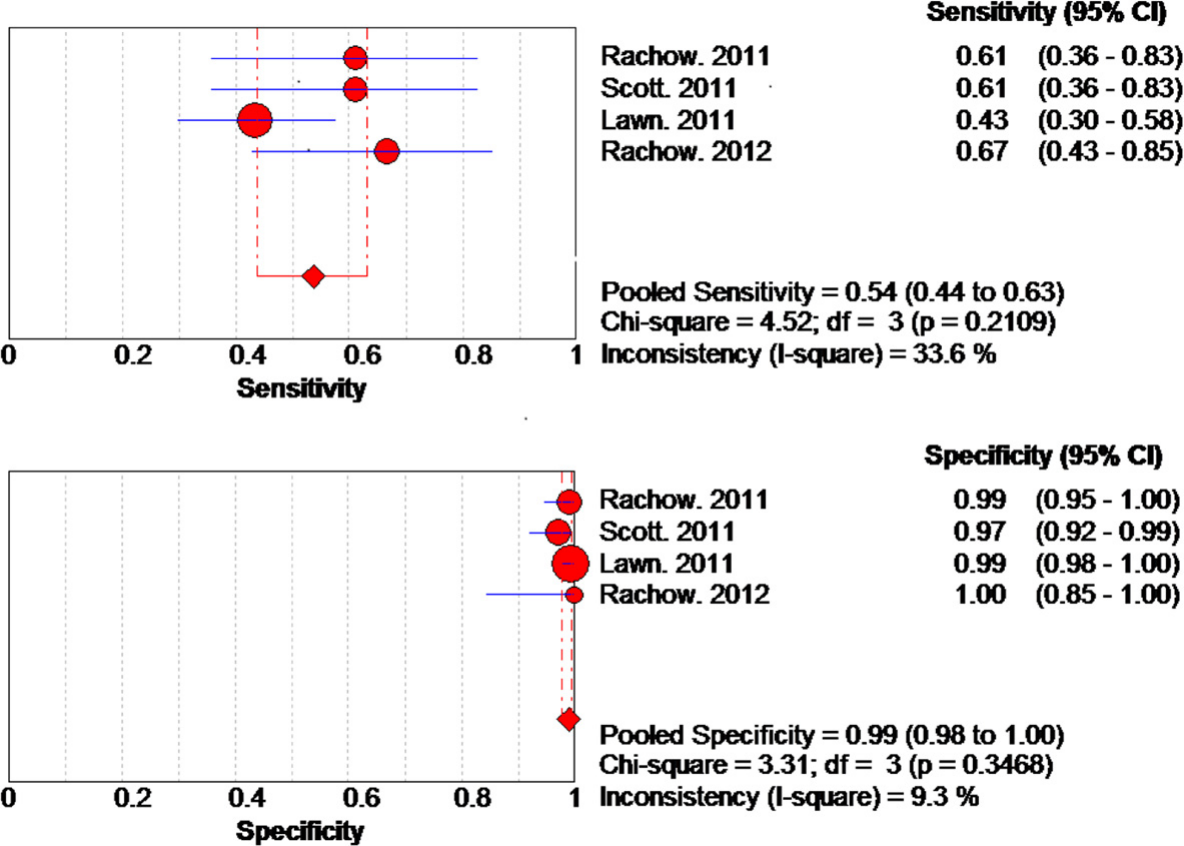
Forest plots of sub-analysis of sensitivity and specificity of GeneXpert.

For the GeneXpert, we focused on publications of studies in settings with HIV prevalence ≥ 30%, a typical value for TB patients from sub-Saharan Africa, where the GeneXpert is expected to be of much benefit due to the high levels of HIV-associated TB [9]. There were four publications, from such high HIV prevalence settings; two from South Africa and two from Tanzania, which we sub- analyzed. The pooled sensitivity of the GeneXpert from these settings was reduced from 67% to 54%, while the specificity remained 99%. These results are presented in Figure 5. For the WHO algorithm, a similar sub-group analysis gave a sensitivity of 65% and a specificity of 55%. We did not perform a similar sub-analysis for the MODS because the publications were inadequate for the analysis. Instead, we performed a sub-group analysis, excluding the outlier study which had reported what the authors decsribed as “ unexplained observed disturbing inconsistencies in results”, when they used the MODS for diagnosis of smear-negative TB [40]. Pooled sensitivity of MODS increased from 73% to 82%, and specificity increased from 91% to 95%.

#### Based on screening tests used

Since FM microscopy is increasingly becoming an alternative to ZN microscopy for diagnosis of TB in several settings [48], we also performed subgroup analysis for studies that used FM versus ZN as screening tests. The sensitivity for GeneXpert for studies that used FM as screening test was 52% and specificity was 98%. For studies that used ZN the sensitivity for GeneXpert was 69% and specificity was 99%. None of the studies evaluating MODS used FM as a screening test, thus a similar sub-analysis was not possible. However, the sensitivity for studies that evaluated MODS using ZN as screening test was 73%, while the specificity was 90%. There were an inadequate number of studies that evaluated WHO 2007 algorithm using FM as a screening test. However, for those studies that used ZN as the screen test, the sensitivity was 60% and the specificity was 61%.

#### Based on patients not completing all elements of the WHO 2007 algorithm

Lastly, since the WHO 2007 algorithm is widely used for diagnosis of smear-negative TB, but in practice few patients complete all the elements of the algorithm before clinicians exclude or initiate treatment for smear-negative TB, we performed a sub-group analysis of the WHO algorithm, excluding the publication that reported performance of the algorithm based on data of those patients that completed all the elements of the algorithm before clinicians decided if to treat or not [8]. The pooled sensitivity of the WHO algorithm reduced from 61% to 57%, while specificity increased marginally from 69% to 70% (data not shown).

### QUADAS results of meta-analyzed publications

**Figure 6:**
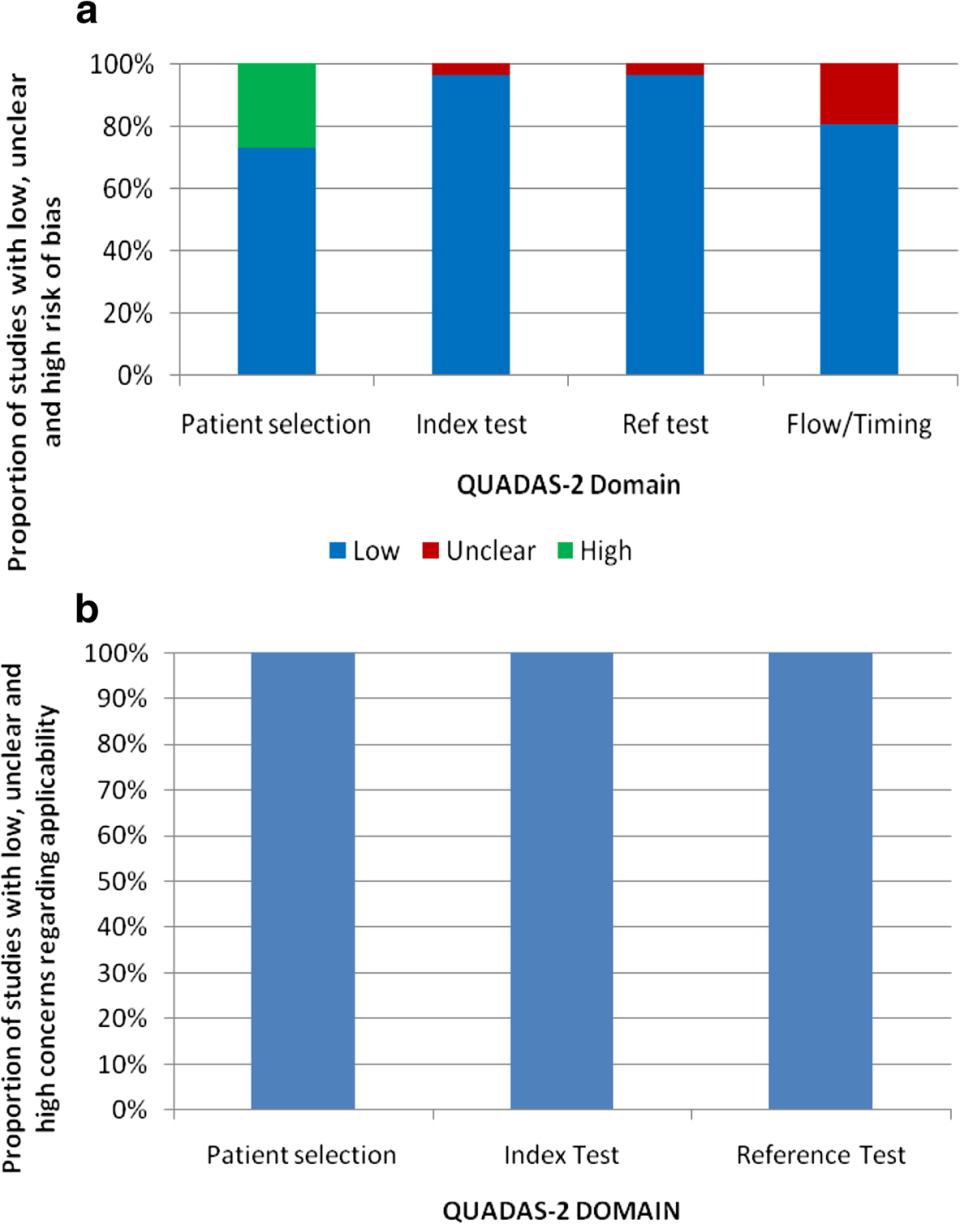
QUADAS-2 Results of (a) risk of bias and (b) concerns on applicability.

Seventeen out of the 24 meta-analyzed publications (70%) had a low risk of bias. Of the publications at risk of bias, six were on GeneXpert, while one was on WHO 2007 algorithm. The source of risk in these publications arose principally from unclear and flow of patients. However, all the publications matched the review questions, and therefore had low concern for applicability. The overall quality of the 24 publications is shown in Figure 6, while the quality of for the individual studies are shown in Table 1.

## Discussion

We set out to compare the accuracy of GeneXpert, MODS and the WHO 2007 algorithm, for diagnosis of SN-PTB by doing a meta-analysis of the published literature. To our knowledge, this is the first study done to compare the accuracy of the three methods for the diagnosis of SN-PTB.

Overall, the MODS had the highest pooled sensitivity of 73%, followed by the GeneXpert with sensitivity of 67% and the WHO 2007 algorithm with sensitivity of 61%. GeneXpert had the highest pooled specificity of 98%, followed by MODS with 91% and the WHO 2007 algorithm with 69%.

There was substantial heterogeneity in the accuracy estimates for all the three tests that we evaluated, with the inconsistence indices (I^2^) ranging from 71% to 90% for sensitivity, and 70% to 93% for specificity. Considering the sROC curves in view of the substantial heterogeneity, the GeneXpert had the highest accuracy for detection of SN-PTB with an area under the curve of a constructed sROC curve (AUC) value of 0.94, followed by MODS with 0.88 and the WHO algorithm with 0.69.

Several reasons can explain the heterogeneity that we observed. These include; variations in the HIV prevalence among study patients and the corresponding variation in the severity of TB disease. Additionally use of either FM or ZN as the screening test including operator/technician performance, type of specimen tested, and differences in the culture methods used as reference test can explain the variability.

Thus, the observed heterogeneity for the GeneXpert could be due to differences in the severity of HIV and the co-morbidities among the patients evaluated, since the test is fully automated after sample processing, requiring no technician involvement. On the other hand, technician performance could be a major factor in the heterogeneity observed for MODS, since inexperienced technicians could confuse artefacts for M.tb cords.

With regard to heterogeneity observed for the WHO 2007 algorithm, few clinicians fully adhere to the algorithm in practice, due to operational difficulties. Therefore, the decision by clinicians if to treat or not for SN-PTB is made variably. For example, of the 4 studies on the WHO 2007 algorithm in our review, only 1 reported results based on full adherence to all the elements of the algorithm [8]. However, full adherence to the algorithm in this study was quite low, ranging from 13% for the rural site to 19% for the urban site. Based on this report, in a best case scenario, the sensitivity of the algorithm is 95% (95% CI; 74%-100%) while specificity is 33% (95% CI; 23%-68%). On the other hand, based on the 3 other reports on the WHO 2007 algorithm, the sensitivity of the algorithm in a real world scenario is 57% (95% CI; 50%- 64%,) while specificity is 70% (95% CI; 66%-73%). The variable access to some of the tests in the algorithm such as chest X-ray could explain the heterogeneity observed for the WHO 2007 algorithm.

Our results of the GeneXpert for diagnosis of SN-PTB are similar to those recently reported by the Cochrane Collaboration® [49]. Both our findings and those by the Cochrane group are however lower than what was reported in another publication, where the authors found sensitivity of GeneXpert for smear-negative PTB to be 75% and specificity 98% [50]. However, it was not clear whether they used the random-effects model for this subgroup analysis in their report. The random-effects model is the recommended analytical approach for meta-analysis since it incorporates heterogeneity among studies as opposed to the fixed-effects model which ignores heterogeneity [51].

Unlike in the report by the Cochrane group, where meta-analysis for the effect of HIV on the diagnostic accuracy of GeneXpert for SN-PTB could not be done, due to the small numbers of publications, in our study we found that the sensitivity of GeneXpert reduced from 67% to 54% while specificity remained unchanged. This finding was based on four studies with HIV prevalence ≥ 30%, an HIV rate which is commonly seen in six of the nine TB high burden countries from sub-Sahara Africa [1].

We used a comprehensive search and selection strategy which has been used before [52]. Further, most information (70%) was from publications which had low risk of bias, while all (100%), had low concerns regarding applicability (Figure 6a and b and Table 3). This implies good internal and external validity of the results in the primary studies. We therefore believe that our findings are robust. In addition any plausible bias is unlikely to alter the results as the confidence intervals for all the tests was narrow.

**Table 3:** QUADAS-2 results of risk of bias and concerns on applicability for each study included in the meta-analysis (n = 24)

		**Risk of bias**				**Applicability concerns**		
**Test**	**Author, Year**	**Patient selection**	**Index test**	**Reference standard**	**Flow and timing**	**Patient selection**	**Index test**	**Reference standard**
Genexpert	Helb [24]	(+)	(+)	(+)	(+)	(+)	(+)	(+)
Genexpert	Malbruny [25]	(+)	(+)	(+)	(+)	(+)	(+)	(+)
Genexpert	Bowles [26]	(- )	( ? )	( ? )	( ? )	(+)	(+)	(+)
Genexpert	Moure [27]	(+)	(+)	(+)	(+)	(+)	(+)	(+)
Genexpert	Marlowe [28]	(+)	(+)	(+)	(+)	(+)	(+)	(+)
Genexpert	Theron [29]	(+)	(+)	(+)	(+)	(+)	(+)	(+)
Genexpert	Rachow [30]	(+)	(+)	(+)	(+)	(+)	(+)	(+)
Genexpert	Scott [31]	(- )	(+)	(+)	(+)	(+)	(+)	(+)
Genexpert	Lawn [32]	(+)	(+)	(+)	(+)	(+)	(+)	(+)
Genexpert	Ioannidis [33]	(- )	(+)	(+)	( ? )	(+)	(+)	(+)
Genexpert	Miller [34]	(- )	(+)	(+)	( ? )	(+)	(+)	(+)
Genexpert	Teo [35]	(- )	(+)	(+)	( ? )	(+)	(+)	(+)
Genexpert	Nicol [36]	(+)	(+)	(+)	(+)	(+)	(+)	(+)
Genexpert	Rachow [37]	(+)	(+)	(+)	(+)	(+)	(+)	(+)
Genexpert	Safianowska [38]	(- )	(+)	(+)	( ? )	(+)	(+)	(+)
MODS	Arias [39]	(+)	(+)	(+)	(+)	(+)	(+)	(+)
MODS	Mashta [40]	(+)	(+)	(+)	(+)	(+)	(+)	(+)
MODS	Shah [41]	(+)	(+)	(+)	(+)	(+)	(+)	(+)
MODS	Ha DT [42]	(+)	(+)	(+)	(+)	(+)	(+)	(+)
MODS	Chaiyasirinroje [43]	(+)	(+)	(+)	(+)	(+)	(+)	(+)
WHO2007	Wilson [44]	(+)	(+)	(+)	(+)	(+)	(+)	(+)
WHO2007	Swai [45]	(+)	(+)	(+)	(+)	(+)	(+)	(+)
WHO2007	Koole [46]	(+)	(+)	(+)	(+)	(- )	(+)	(+)
WHO2007	Alamo [8] Rural site	(-)	(+)	(+)	(+)	(-)	(+)	(+)
WHO2007	Alamo [8] Urban site	(-)	(+)	(+)	(+)	(+)	(+)	(+)

(+) = low. (-) = High. (?) = Unclear.

### Limitations

Our study had the following limitations: There were few publications on MODS and the WHO2007 algorithm on diagnosis of SN-PTB. Moreover a substantial number of the publications on these two tests had to be excluded due to lack of reported data to compute sensitivity and specificity of the tests for diagnosis of SN-PTB. This included 2 large landmark studies on GeneXpert for the same reason [22,23]. The negative or positive influence of these studies on the pooled accuracy of the tests could therefore not be established. Further, although there was substantial heterogeneity across all studies for the three diagnostic methods, we did not perform a meta-regression analysis to investigate the effects of the various characteristics associated with the observed heterogeneity. However, our primary aim was not to explore the factors that may be accountable for the differences among studies. Besides, to achieve reliable conclusions from such an investigation, one would need to pre-specify the protocol of the review since explorations of heterogeneity that are devised after heterogeneity is identified cannot be conclusive. We did not also assess publication bias of the studies which we meta-analyzed. This was because there were few studies on MODS and the WHO algorithm for such analysis [53]. In addition, despite its cited advantages (such as being free and user friendly), the meta-disc software which we used in our analysis is limited in some statistical tests including the Egger’s test and Begg’s tests that are recommended for assessing publication bias.

## Conclusions

The GeneXpert, MODS, and the WHO algorithm have moderate to high accuracy for the diagnosis of SN-PTB. However, the accuracy of the tests is extremely variable. The setting and context under which the tests are conducted in addition to several other factors could explain this variability. There is therefore need to investigate these factors further. The information from these studies would inform the adoption and placement of these new tests.

## Competing interests

The authors declare that they have no competing interests.

## Authors’ contributions

All the authors planned and designed the study. SW and FB: retrieved and reviewed the study reports, summarized and analysed the data, and prepared the manuscript. MH and ADC: retrieved some of the study reports and critically revised the manuscript versions. MJ and SH: Critically revised the manuscript versions. All authors read and approved the final manuscript.

## Pre-publication history

The pre-publication history for this paper can be accessed here:

http://www.biomedcentral.com/1471-2334/13/507/prepub
